# Tamoxifen use and acute pancreatitis: A population-based cohort study

**DOI:** 10.1371/journal.pone.0173089

**Published:** 2017-03-14

**Authors:** Fan-Gen Hsu, Yow-Wen Hsieh, Ming-Jyh Sheu, Che-Chen Lin, Cheng-Li Lin, Chung Y. Hsu, Chang-Yin Lee, Mei-Yin Chang, Kuang-Hsi Chang

**Affiliations:** 1 School of Pharmacy, China Medical University, Taichung, Taiwan; 2 Department of Pharmacy, China Medical University Hospital, Taichung, Taiwan; 3 Healthcare Service Research Center (HSRC), Taichung Veterans General Hospital, Taichung, Taiwan; 4 Management Office for Health Data, China Medical University Hospital, Taichung, Taiwan; 5 Graduate Institute of Clinical Medical Science, China Medical University, Taichung, Taiwan; 6 College of Medicine, The School of Chinese Medicine for Post Baccalaureate, I-Shou University (Yancho Campus), Kaohsiung, Taiwan; 7 Department of Chinese Medicine, E-DA Hospital, Kaohsiung, Taiwan; 8 Department of Medical Laboratory Science and Biotechnology, School of Medical and Health Sciences, Fooyin University, Kaohsiung, Taiwan; 9 Institute of Biomedical Sciences, China Medical University, Taichung, Taiwan; University of South Alabama Mitchell Cancer Institute, UNITED STATES

## Abstract

**Background:**

Several case reports have indicated that tamoxifen induced acute pancreatitis (AP); but no pharmacoepidemiological data support the claim. Therefore, we investigated whether tamoxifen use is correlated with the risk of AP in patients with breast cancer.

**Methods:**

This population-based cohort study used the Taiwan National Health Insurance Research Database. A cohort of 22 005 patients aged ≥20 years with breast cancer from January 1, 2000 to December 31, 2009 was identified and the date of cancer diagnosis was set as the index date. The end point was developing AP during the follow-up. Hazard ratios (HRs) and 95% confidence intervals (CIs) were evaluated to determine the correlation between the risk of AP and tamoxifen use. Because the drug use varied over time, it was measured as a time-dependent covariate in the Cox proportional hazard model. The same approaches were applied in PS-matched cohorts.

**Results:**

After adjustment for covariates and medication use including fluorouracil and doxorubicin, the risk of AP was not significant between tamoxifen users and tamoxifen nonusers (adjusted HR = 0.94, 95% CI = 0.74–1.19) in the non-matching cohorts. The results revealed no dose–response trend between tamoxifen use and the risk of AP (adjusted HR = 0.98, 95% CI = 0.96–1.00). The comorbidities DM and gallstones were associated with a significantly increased risk of AP. Similar trends were observed in PS-matched cohorts.

**Conclusions:**

No significant correlation was observed between tamoxifen use and the risk of AP in patients with breast cancer.

## Introduction

Breast cancer has the highest incidence and mortality among all cancers in females worldwide, accounting for an estimated 1.7 million cases (25% of all new cancer cases) and 521 900 deaths (15% of total cancer-related deaths) in 2012 [1]. Tamoxifen is a selective estrogen receptor (ER) modulator. Its estrogen antagonist activity has been widely used to treat ER-positive breast cancer for approximately 4 decades [2]. Tamoxifen therapy for 5 years can reduce the annual mortality rate by 31% and prevent cancer over the next 20 years [3, 4]. Additionally, tamoxifen acts as an estrogen agonist in the bones, the endometrium, the coagulation system, and lipoprotein metabolism and exerts beneficial effects by maintaining the bone density and lowering the cholesterol level [2, 5] Tamoxifen is generally a safe and well-tolerated drug, but has certain adverse effects such as endometrial cancer, thromboembolism, and menopausal symptoms of clinical concern [2]. Moreover, 9 case reports have described tamoxifen-induced AP with severe hypertriglyceridemia and one event of death [6, 7].

AP is an acute inflammation of the pancreas and is the leading cause of hospitalization among all gastrointestinal disorders in the United States (274 119 discharges were reported in 2009; USD 2.6 billion per year are spent on in-hospital costs) and several other countries [8, 9]. AP-associated inflammation may expand to local and remote extra pancreatic tissues. It is a major public health concern owing to its significant potential mortality and morbidity, with an increasing rate of incidence and hospitalization [8–10]. In addition, several routinely prescribed drugs increase the risk of AP; medication use was reported as the cause of 0.1%– 5% of AP cases [11, 12]. Increasing time trends were reported in a proportion of first-attack AP patients on pancreatitis-related drug therapy from 2003 to 2012 [11].

Based on the WHO database from 1968 to 1993, 525 different drugs were suspected to cause AP [13]. However, overdependence on spontaneous adverse drug reactions or case reports is likely to exaggerate the true risk, which might therefore remain controversial without deriving any conclusion regarding the correlations between drugs and the risk of AP [13, 14]. Thus, the aforementioned case reports have yielded uncertain results regarding the association between tamoxifen use and the risk of AP. To address this clinical concern, we designed a formal pharmacoepidemiological study with a cohort design using nationwide population-based data to investigate whether tamoxifen use is correlated with the risk of AP in Taiwanese patients with breast cancer.

## Methods

### Data sources

The National Health Insurance Research Database (NHIRD) is a database containing claims data from the Taiwan National Health Insurance program (Taiwan NHI) and is managed by the National Health Research Institutes (NHRI). The Taiwan NHI was organized by combining 13 then-existing health programs into a compulsory nationwide and single-payer health program in 1995. The Taiwan NHI covered more than 99% of 23 million residents in Taiwan [15]. The NHIRD comprises claims data including a registry of beneficiaries, disease records, a registry of drug prescriptions, and records on other medical services. Diagnoses of cancer were identified in the registry of catastrophic illnesses, and comorbidity data were collected from inpatient and outpatient files. The disease records containing the registry of catastrophic illnesses and inpatient and outpatient files were based on the International Classification of Diseases, Ninth Revision, Clinical Modification (ICD-9-CM). To protect the privacy of insurants, the NHRI encrypts identification numbers before releasing the database to researchers. The study was approved by the Ethics Review Board of China Medical University (CMUH104-REC2-115).

### Study population

We selected patients with new-onset breast cancer (ICD-9-CM code 174) who were aged ≥20 years during 2000 and 2009 and assigned the index date as the date of cancer diagnosis. We excluded patients with a history of AP (ICD-9-CM code 577.0), chronic pancreatitis (ICD-9-CM code 577.1), and pancreatic cancer (ICD-9-CM code 157) before the index date.

Tamoxifen use (Anatomical Therapeutic Chemical [ATC] code L02BA01) was analyzed in this study. To standardize the dose of drug use, all drugs were categorized according to the ATC codes, and the accumulated defined daily doses (DDDs; tamoxifen 1DDD = 20mg) [16] prescribed during the follow-up period were calculated for each patient. Because the drug use varied with time, information on drug prescription was collected every year after the index date.

Comorbidity was one of the confounding factors considered in the present study. Patients with comorbidity were those who had a history of comorbidity before the index date. Alcohol-related disease (ARD, ICD-9-CM codes 291, 303, 305, 571.0, 571.1, 571.2, 571.3, 790.3, and V11.3), diabetes mellitus (DM, ICD-9-CM code 250), hepatitis B virus infection (HBV, ICD-9-CM codes V02.61 and 070.20–070.33), hepatitis C virus infection (HCV, ICD-9-CM codes V02.62, 070.41, 070.44, 070.51, and 070.54), gallstones (ICD-9-CM code 574), hypertriglyceridemia (ICD-9-CM code 272.1), obesity (ICD-9-CM code 278), hyperlipidemia (ICD-9-CM code 272), coronary artery disease (CAD, ICD-9-CM code 410–414), chronic obstructive pulmonary disease (COPD, ICD-9-CM code 491, 492, 496), and asthma (ICD-9-CM code 493) were analyzed in this study and fluorouracil, and doxorubicin medication was also considered at endpoints. The accuracy of the diagnoses of the comorbidity assessed in the present study has been validated in previous studies [9, 17–19].

Additional tamoxifen used and Propensity Score (PS) -matched non-tamoxifen used cohorts were formed. Logistic regression was used to estimate the probability of the treatment assignment by calculating the PS. Baseline variables for calculating the PS included the index year, age, medication use including fluorouracil and doxorubicin, and comorbidities including ARD, DM, HBV, HCV, gallstones, hypertriglyceridemia, obesity, hyperlipidemia, CAD, COPD, and asthma.

All patients were followed up from the index date until the patient withdrew from the NHI, AP occurrence, or December 31, 2011.

### Statistical analysis

Two statistical methods were used to compare the risk of acute pancreatitis between the tamoxifen users and tamoxifen nonusers groups. One method employed a non-matching design, and the other method employed a PS-matching design. The means and standard deviations were used to present continuous variables, and numbers and percentages were used to present categorical variables. To evaluate the difference between patients who developed AP and those who did not, we applied the *t* test for continuous variables and chi-square test for categorical variables. Because patients with breast cancer may not have taken pharmacy regularly during the study period and this may lead to an overestimation of the effect of the drug, we used the Cox proportional hazard model with time-dependent exposure covariates to estimate the risk of AP to reduce this bias. The hazard ratios (HRs) and 95% confidence intervals (CIs) of AP in tamoxifen users and tamoxifen nonusers were evaluated. The SAS software (Version 9.4, SAS Institute, Cary, NC, USA) was used for data management and statistical analysis. A 2-sided *P* value < .05 was considered statistically significant.

## Results

Table 1 lists the baseline characteristics, comorbidity statuses and treatment of the breast cancer patients in all tamoxifen users or tamoxifen nonusers patients and the PS-matched cohorts. In non- matching cohorts, the mean age in the tamoxifen users group was 51.4 ± 11.6 years and 53.4 ± 12.0 years in the tamoxifen nonusers group, respectively. DM, HCV, hyperlipidemia, CAD, COPD, and asthma were more prevalent in the tamoxifen nonusers group than in the tamoxifen users group. There’s significant difference of medication history of fluorouracil use between the two groups (54.5% vs. 59.7%; *P*<0.001). No significant differences were observed for age, comorbidity and medication statuses between the tamoxifen users and the PS-matched tamoxifen nonusers groups.

**Table 1:** Distributions of demographic variables and comorbidities in breast cancer patients with and without propensity score matching.

				Propensity Score Matched		
	Without Tamoxifen used	With Tamoxifen used	p	Without Tamoxifen used	With Tamoxifen used	p
n	22005	40484		22005	22005	
Age at baseline, years (SD)^†^	53.4(12.0)	51.4(11.6)	<0.001	53.4(12.0)	53.4(12.3)	0.93
Comorbidity						
ARD	109(0.50)	234(0.58)	0.18	109(0.50)	101(0.46)	0.58
DM	2785(12.7)	4467(11.0)	<0.001	2785(12.7)	2835(12.9)	0.48
HBV	679(3.09)	1214(3.00)	0.54	679(3.09)	663(3.01)	0.66
HCV	332(1.51)	498(1.23)	0.004	332(1.51)	333(1.51)	0.97
Gallstone	1003(4.56)	1736(4.29)	0.12	1003(4.56)	1008(4.58)	0.91
Hypertriglyceridemia	316(1.44)	549(1.36)	0.41	316(1.44)	320(1.45)	0.87
Obesity	409(1.86)	715(1.77)	0.41	409(1.86)	426(1.94)	0.55
Hyperlipidemia	5345(24.3)	9107(22.5)	<0.001	5345(24.3)	5382(24.5)	0.68
CAD	3564(16.2)	5971(14.8)	<0.001	3564(16.2)	3556(16.2)	0.92
COPD	2365(10.8)	3992(9.86)	0.001	2365(10.8)	2335(10.6)	0.64
Asthma	1976(8.98)	3390(8.37)	0.01	1976(8.98)	1958(8.90)	0.76
Treatment						
Fluorouracil	11994(54.5)	24186(59.7)	<0.001	11994(54.5)	11840(53.8)	0.14
Doxorubicin	3934(17.9)	7427(18.4)	0.15	3934(17.9)	3859(17.5)	0.35

^†^*t* test.

Abbreviations: ARD: alcohol-related disease; DM: diabetes mellitus; HBV: hepatitis B virus infection; HCV: hepatitis C virus infection.

After adjustment for age, ARD, DM, HBV, HCV, gallstones, hypertriglyceridemia, obesity, hyperlipidemia, CAD, COPD, and asthma and medication use including fluorouracil and doxorubicin, the risk of AP was not significant between tamoxifen users and tamoxifen nonusers (adjusted HR = 0.94, 95% CI = 0.74–1.19) (Table 2) in the non-matching cohorts. The results revealed no dose–response trend between tamoxifen use and the risk of AP (adjusted HR = 0.98, 95% CI = 0.96–1.00). The comorbidities DM and gallstones were associated with a significantly increased risk of AP. Similar trends were observed in PS-matched cohorts (S1 Table).

**Table 2:** Crude and adjusted hazard ratios and 95% confidence intervals of the incidence of acute pancreatitis in tamoxifen users and nonusers according to a time-dependent Cox regression model without propensity score matching.

Variable	Unadjusted HR (95% CI)	Adjusted HR (95% CI)
Tamoxifen used (Yes versus No)	0.87 (0.69–1.10)	0.94 (0.74–1.19)
Increased dose of Tamoxifen, per 100 DDD	0.98 (0.96–1.00)	0.98 (0.96–1.00)
Age at baseline		
<45	ref	ref
45–64	1.50(1.12–2.02)	1.21 (0.89–1.64)
≥65	4.11(2.99–5.65)	2.24 (1.54–3.24)
ARD (Yes versus No)	2.08(0.67–6.49)	1.56 (0.50–4.88)
DM (Yes versus No)	2.70(2.10–3.48)	1.63 (1.23–2.16)
HBV (Yes versus No)	1.30(0.71–2.37)	1.15 (0.62–2.10)
HCV (Yes versus No)	2.24(1.11–4.53)	1.28 (0.63–2.61)
Gallstone (Yes versus No)	4.38(3.24–5.93)	3.19 (2.33–4.35)
Hypertriglyceridemia (Yes versus No)	2.25(1.16–4.36)	1.10 (0.56–2.18)
Obesity (Yes versus No)	0.57(0.18, 1.79)	2.22(0.71, 6.94)
Hyperlipidemia (Yes versus No)	2.08(1.67, 2.61)	0.85(0.65, 1.10)
CAD (Yes versus No)	2.59(2.05, 3.28)	0.67(0.51, 0.88)
COPD (Yes versus No)	1.47(1.07, 2.02)	1.09(0.78, 1.54)
Asthma (Yes versus No)	1.31(0.91, 1.87)	1.10(0.75, 1.62)
Treatment		
Fluorouracil (Yes versus No)	0.62(0.50, 0.78)	1.25(1.00, 1.56)
Doxorubicin (Yes versus No)	0.71(0.51, 0.98)	0.94(0.74, 1.19)

The Cox proportional hazard model was adjusted for age, ARD, DM, HBV, HCV, gallstones, and hypertriglyceridemia, obesity, hyperlipidemia, CAD, COPD, and asthma, and treatment of fluorouracil and doxorubicin. Abbreviations: ARD: alcohol-related disease; DM: diabetes mellitus; HBV: hepatitis B virus infection; HCV: hepatitis C virus infection; CAD: coronary artery disease; COPD: chronic obstructive pulmonary disease. HR: hazard ratio; CI: confidence interval; DDD: defined daily dose.

## Discussion

In this present study, we observed no statistically significant correlation between tamoxifen use and the risk of AP, but detected a marginal reduction in the risk of AP after tamoxifen use (adjusted HR = 0.93, 95% CI = 0.73–1.18). We did not observe any dose–response trend between tamoxifen use and the risk of AP (adjusted HR = 0.98, 95% CI = 0.96–1.00). To our knowledge, the present study is the first population-based cohort study to examine the correlation between the use of tamoxifen and the risk of AP, and no other data could be compared with its results. We reviewed related articles to validate the biological plausibility of the finding as follows:

First, tamoxifen exerts estrogen agonist activities on serum lipid metabolism. Kim et al and Singh et al reported that all nine patients with tamoxifen-induced AP had hypertriglyceridemia (as an intermediary) [6, 7, 20]. Moreover, in a 9-year follow-up cohort study, Lim et al used the NHIRD to demonstrate that tamoxifen use in patients with breast cancer was associated with a decreased risk of hyperlipidemia; however, the researchers failed to mention whether tamoxifen use was associated hypertriglyceridemia or the risk of AP [21]. Subsequently, our review of studies analyzing the effect of tamoxifen use on serum hypertriglyceridemia concentration revealed the following important observations: Twelve case reports on tamoxifen-induced severe hypertriglyceridemia (serum triglycerides >1000 mg/dL) demonstrated that only 4 patients showed induced pancreatitis and most of these patients had a personal or family history of hyperlipidemia [22]. In addition, a retrospective study assessed the effect of tamoxifen use on changes in triglyceride levels and suggested that triglyceride levels significantly increased only in patients with a disturbed triglyceride-rich lipoprotein metabolism (susceptible patients) [23]. Furthermore, there was evidence that tamoxifen can induce hypertriglyceridemia in susceptible patients [22]. Because the NHIRD does not record the status of triglyceride-rich lipoprotein metabolism, we could confirm that tamoxifen use is no significantly correlated with AP in patients with breast cancer, but we could not confirm whether susceptible patients carry an insignificant risk (HR) of AP or whether non susceptible patients carry no risk of AP. Additional prospective studies are warranted to address this concern.

Second, tamoxifen, in addition to its partial estrogen agonist activity on lipoprotein metabolism, can induce severe hypertriglyceridemia and pancreatitis [2, 22]. Because of its estrogen antagonist activity through competitive inhibition at the estrogen binding sites on ERs, the drug is used in ER-positive breast cancer [2]. However, whether the estrogen antagonist activity exerts a protective effect on the pancreas remains unknown. Therefore, we researched some relevant literature to address this concern. A review article reported that ERs and estrogen-binding proteins are present in the healthy human pancreas [24]. Tamoxifen, when combined with other chemotherapeutics, was effective in treating pancreatic cancer in phase II trials, independent of the hormone receptor status [25,26]. Moreover, another study reported that tamoxifen exerted a cytotoxic effect against tumor cells of the pancreas, independent of the hormone receptor status [27]. All these studies indicate the effectiveness of tamoxifen in pancreatic cancer cells, with no mention of the protective effect of the drug on the pancreas and its association with a decreased risk of AP. The estrogen antagonist activity of tamoxifen may override the estrogen agonist activity, and a protective effect was demonstrated in some cases. This partly explains why we observed a marginal decrease with no statistical significance in the risk of AP after tamoxifen use. However, additional prospective studies are warranted to address the concern regarding the correlation between the estrogen antagonist activity of tamoxifen use and the risk of AP.

Alcohol is a known cause of acute and chronic pancreatitis. We used alcohol-related diseases (including alcoholic psychoses, alcohol dependence syndrome, alcohol abuse, alcoholic fatty liver, acute alcoholic hepatitis, alcoholic cirrhosis, alcoholic liver damage, excessive blood level of alcohol, and personal history of alcoholism) instead of alcohol use. Unexpectedly, on the bases of the multivariable analysis, alcohol-related diseases (adjusted HR = 1.56, 95% CI, 0.50–4.88) was not associated with AP. (Table 2) There might be the difficulty of documenting heavy alcohol use but prior to the onset of alcoholic liver disease. The ICD-9 codes document alcohol-related diseases, but does not necessarily include regular alcohol use prior to a presentation of alcohol disease. A prospective research need to clarify this issue.

Our study has several strengths. The NHIRD database, its extensiveness, and its ability to be used in addressing several clinical questions were extensively reviewed by Hsing et al. [28]. Breast cancer is considered a catastrophic illness by the NHI, and most medical copayments and drug cost-sharing are exempted. Therefore, tamoxifen use does not tend to change with the social class, reducing the selection bias in our study. Although the use of AP diagnoses coded according to the ICD-9-CM was documented by Lai et al and Hung et al in well-known lituretures, this claims dataset lack of serum amylase (or lipase) level, and abdominal imaging (eg, computed tomography scan) findings to confirm the AP diagnoses [17, 19]. The concern about misclassification of AP diagnosis might be occurred with a low probability, because the disease diagnosis without valid supporting clinical findings may be considered a medical fraud by NHI with a penalty of 100-fold of the payment claimed by the treating physician or hospital. However, the high coverage rate of the Taiwan NHI and high accessibility to Taiwan’s medical care service might make those omissions negligible. Therefore, the AP diagnosis by ICD-9 code in this study should be reliable. Finally, data on tamoxifen use did not depend on interviews and questionnaires, thus preventing any self-reporting errors and avoiding recall bias.

Nonetheless, some limitations should be considered when interpreting our study data. First, the NHIRD does not provide detailed information on potential covariates such as lifestyle habits, diet, and obesity (body mass index, waist circumference data) [19]. However, our study patients were limited to patients with breast cancer, thus partially reducing selection bias. The NHIRD data covers more than 99% of residents in Taiwan and the reimbursement policy is universal. Thus, it was unlikely for these covariates to differ significantly between the drug users and nonusers. Second, partial selection bias might have occurred because the tamoxifen users were more closely monitored and thus had a higher likelihood of being diagnosed with AP or undergoing serum triglyceride level evaluation and correction. However, our results demonstrated a null association, with a marginal decrease in the risk of AP after tamoxifen use. Third, we could not determine whether the patients actually consumed tamoxifen, because the NHIRD simply records the prescriptions rather than the actual dose consumed by patients. Because tamoxifen is usually prescribed to patients diagnosed with breast cancer, the prescription rate and patient compliance were probably relatively high owing to the low risk–benefit ratio of the drug. In addition [2], in a large-scale population-based study, the effects of tamoxifen prescription noncompliance can be neglected. Fourth, genetics may be a factor that predisposes people to AP [14, 18]. Despite using a large sample, information on serum triglyceride levels and genetic testing results was unavailable for our dataset; further, these findings only represent the Taiwanese ethnic group [19].

In conclusion, no significant correlation between tamoxifen use and the risk of AP was observed in patients with breast cancer. According to the foregoing discussion, additional prospective studies are warranted to address the concerns.

## Supporting information

S1 Table Crude and adjusted hazard ratios and 95% confidence intervals of the incidence of acute pancreatitis in tamoxifen users and nonusers according to a time-dependent Cox regression model with propensity score matching. (DOCX)
