# Supplementary material for: Tamoxifen use and acute pancreatitis: A population-based cohort study
Source: PLoS One. 2017 Mar 14;12(3):e0173089. doi: 10.1371/journal.pone.0173089 (PMC5349648; doi:10.1371/journal.pone.0173089)
Supplement: S1 Table — (DOCX) [file pone.0173089.s001.docx]

Table 3: **Crude and adjusted hazard ratios and 95% confidence intervals of the incidence of acute pancreatitis in** tamoxifen users and nonusers according to a time-dependent Cox regression model with propensity score matching.

| Variable | Unadjusted HR (95% CI) | Adjusted HR (95% CI) |
| --- | --- | --- |
| Tamoxifen used ( Yes versus No) | 0.94(0.70, 1.24) | 0.95(0.71, 1.26) |
| Increased dose of Tamoxifen, per 100 DDD | 0.99(0.96, 1.01) | 0.98(0.96, 1.01) |
| Age at baseline |  |  |
| <45 | ref | ref |
| 45-64 | 2.25(1.49, 3.42) | 1.80(1.17, 2.75) |
| ≥65 | 5.84(3.79, 9.00) | 3.20(1.97, 5.20) |
| ARD ( Yes versus No) | 1.07(0.15, 7.62) | 0.77(0.11, 5.54) |
| DM ( Yes versus No) | 3.01(2.27, 4.00) | 1.78(1.30, 2.44) |
| HBV ( Yes versus No) | 1.48(0.76, 2.88) | 1.34(0.68, 2.63) |
| HCV ( Yes versus No) | 2.35(1.11, 4.98) | 1.30(0.61, 2.79) |
| Gallstone ( Yes versus No) | 4.87(3.48, 6.82) | 3.46(2.44, 4.89) |
| Hypertriglyceridemia ( Yes versus No) | 2.00(0.89, 4.49) | 0.91(0.40,2 .09) |
| Obesity ( Yes versus No) | 0.76(0.24, 2.36) | 1.69(0.54, 5.31) |
| Hyperlipidemia ( Yes versus No) | 2.21(1.70, 2.86) | 0.85(0.63, 1.15) |
| CAD ( Yes versus No) | 2.58(1.96, 3.39) | 0.73(0.54, 1.00) |
| COPD ( Yes versus No) | 1.58(1.10, 2.26) | 1.06(0.72, 1.56) |
| Asthma ( Yes versus No) | 1.39(0.92, 2.09) | 1.07(0.70, 1.65) |
| Treatment |  |  |
| Fluorouracil ( Yes versus No) | 0.63(0.49, 0.81) | 1.20(0.92, 1.57) |
| Doxorubicin ( Yes versus No) | 0.65(0.44, 0.97) | 1.20(0.81, 1.79) |

The Cox proportional hazard model was adjusted for age, ARD, DM, HBV, HCV, gallstones, and hypertriglyceridemia, obesity, hyperlipidemia, CAD, COPD, and asthma, and treatment of fluorouracil and doxorubicin.

Abbreviations: ARD: alcohol-related disease; DM: diabetes mellitus; HBV: hepatitis B virus infection; HCV: hepatitis C virus infection; CAD: coronary artery disease; COPD: chronic obstructive pulmonary disease.

HR: hazard ratio; CI: confidence interval; DDD: defined daily dose.
